# Bevacizumab-Induced Geographic Tongue

**DOI:** 10.5826/dpc.1103a43

**Published:** 2021-07-08

**Authors:** Marta Martinez-Garcia, Jorge Roman-Sainz, Nicolás Silvestre-Torner, Sergio Tabbara Carrascosa

**Affiliations:** Dermatology Service. Severo Ochoa University Hospital, Leganés, Madrid

**Keywords:** Bevacizumab, geographic tongue, self-solving, infiltrating ductal carcinoma, vascular endothelial growth factor protein

## Introduction

Geographic tongue condition, or benign migratory glossitis, is a self-limited and often recurrent process, which occurs in around 2.5%. of the general population. The diagnosis is clinical and is characterized by the presence of atrophic areas located on the back and lateral edges of the tongue, caused by the absence of filiform papillae. Its cause is unknown, it is associated with multiple systemic diseases and occasionally with drug use.

## Case Presentation

Here we present a case of a 43-year-old patient diagnosed with infiltrating Ductal Carcinoma. 1 year later, he presented tumor recurrence and treatment was started with capecitavin (1500 mg/m2 for 14 days) and bevacizumab (15 mg/m2 every 21 days), obtaining complete remission. In the last 12 months he underwent maintenance therapy with bevacizumab (15 mg/m2/21 days). In the last quarter of the last 12 months the patient reported the appearance of migratory lesions located on the back of the tongue, which evolved in outbreaks and were accompanied by pain and stinging with the ingestion of certain foods. The patient reported no clear relationship with the administration of bevacizumab.

**Figure 1:**
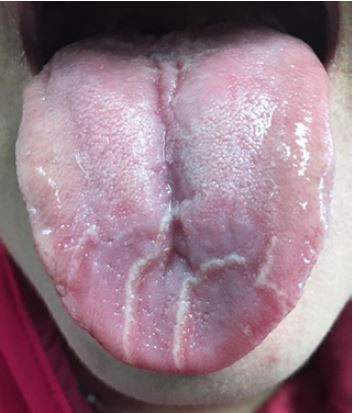
Atrophic plaques surrounded by a keratotic border

**Figure 2:**
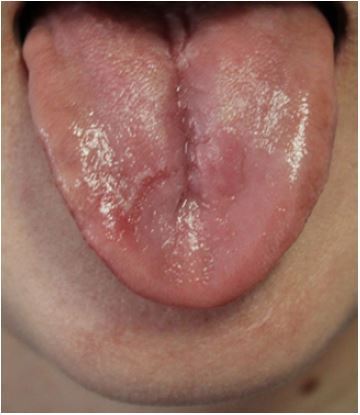
Improvement of lesions

On the first examination, several atrophic plaques were observed, surrounded by a slightly elevated, white, keratotic border, arranged in a concentric fashion (Figure 1). Following 2 follow-up months, an additional examination was performed. A notable spontaneous improvement of the lesions was observed (Figure 2).

## Conclusions

The association of the geographic tongue condition with the use of bevacizumab, whose target is the vascular endothelial growth factor protein A (VEGF-A), has recently been described [1]. VEGF-A activity blockade could impede the normal reparative capacity of the epithelium at the level of the lingual mucosa, which could explain the development of these lesions. The increasing use of this type of drug as a treatment for multiple tumors might facilitate the appearance of new cases of geographic tongue. An in-depth knowledge of the recently described association of the geographic tongue condition following bevacizumab use will prevent misdiagnosis and unnecessary treatment in these patients, who are often immunosuppressed.
